# Feasibility and preliminary effects of non-immersive virtual reality motor-cognitive treadmill training in older Veterans: a single-arm pilot study

**DOI:** 10.1186/s12877-025-06914-5

**Published:** 2026-01-15

**Authors:** Susan S. Conroy, Alyssa Stookey, Brock A. Beamer, John D. Sorkin

**Affiliations:** 1 Baltimore VA Medical Center Geriatric Research, Education and Clinical Center, Veterans Health Administration, Baltimore, MD USA; 2 Department of Physical Therapy and Rehabilitation Science, University of Maryland School of Medicine, Baltimore, MD USA; 3 Department of Medicine, Division of Gerontology, Geriatrics and Palliative Medicine, University of Maryland School of Medicine, Baltimore, MD USA

**Keywords:** Non-immersive virtual reality, Virtual reality, Falls, Ageing, Balance, Dual-task training, Fall prevention

## Abstract

**Background:**

Daily life involves the successful completion of dual task walking activities and is a dynamic integration of motor and cognitive skills to meet environmental and postural demands to avoid a fall. Older Veterans face greater cognitive decline risk, greater comorbidities, and poorer health than non-veterans and have a higher age-related fall risk. Non-immersive VR motor-cognitive treadmill (VR-TM) training can improve balance, walking, and fall risk in older adults but has not been studied in older Veterans and has been largely studied in neurological populations. This pilot study proposed to investigate the acceptance and feasibility of using VR-TM among older Veterans with a history of falls or documented fall risk.

**Methodology:**

Thirty community-dwelling older Veterans aged 65-to-88 years (26 male, 4 female) consented and twenty-three completed this single-arm pilot study. Participants received 14 treatment sessions over 7-weeks using a VR-TM system to walk over virtual obstacles on a treadmill. Balance performance, walking endurance, quality of life (QOL), fear of falling and balance confidence were assessed pre- and post-training. A satisfaction survey was conducted post-training. Descriptive statistics examined fall incidence prior to training and over a six-month retention. Paired t-test and Wilcoxon signed-ranked test performed on the twenty-three completers examined longitudinal change at significance level *P* = 0.05.

**Results:**

The Mini Balance Evaluation Systems Test (Mini-BESTest) had a statistically significant change (20.9 ± 4.20; 23.13 ± 3.03; *P* = 0.01) and six participants had a 4-point improvement reflective of a minimal clinically important difference (MCID). The Activities-specific Balance Confidence scale improved post-training (65.18 ± 21.68; 73.04 ± 18.40; *P* = 0.02) and four participants had a > 18% (MCID) confidence gain. VR-TM training was a feasible intervention in this population and twenty-three out of the thirty consented Veterans (76.6%) completed the protocol. Ninety-four percent of these completers would recommend the VR-TM training to a friend. No significant change was seen in walking performance, walking endurance, QOL outcomes or fall incidence.

**Conclusions:**

The VR-TM training was well accepted and feasible in a small heterogenous cohort of older Veterans with complex comorbidities. Larger randomized trials will be needed to confirm its benefits to balance performance, dual task ability and fall prevention.

**Trial registration:**

This small, non-randomized, non-blinded single-arm feasibility pilot with restricted enrollment to patients at a single medical center was not posted to ClinicalTrials.gov.

## Introduction

Falls threaten independence and are the leading cause of injury among adults aged 65 and older [1]. Approximately 30% of older adults fall each year resulting in an estimated cost of $50 billion in healthcare expenditures [2]. Daily life involves successful coordination of cognitive and motor skills for dual task activities such as walking and talking, texting, or carrying an object. To avoid a fall, one must execute internally generated task goals while simultaneously focusing attention and motor responses on environmental demands and gait stability [3]. This coordination can be difficult as one ages due to changes in musculoskeletal function, balance, postural stability and executive function [4, 5]. Age-related fall risk in men is evidenced by increased reaction times, reduced balance, decreased mobility and decreased strength in their sixties and a decline in ankle flexibility by their seventies [4]. Older Veterans account for 49% of the 16.5 million Veteran population and are predominately male [6]. This group has a higher fall risk profile due to greater comorbidities, poorer health [7], and a greater risk of age-related cognitive decline than non-veterans [8]. The interrelationship of cognitive and motor performance on fall risk is well established [9, 10] but, unfortunately, fall prevention exercise programs (FPEP) largely ignore their interdependence and focus on individual risk factors separately [11]. Several National Council on Aging FPEP (e.g., Otago, Stepping On) reduce falls by 30–35% but don’t emphasize motor-cognitive or dual task training and are largely studied in women [12]. This approach is insufficient and fall-related deaths among older adults are increasing yearly [1]. The complexity of everyday walking and the unique predisposing physical and cognitive risk factors of our aging Veterans necessitates a new approach that incorporates physical and cognitive training in the context of dual task walking.

Technologies, such as non-immersive virtual reality (VR) can automate motor-cognitive training in a safe environment that mimics real-world scenarios [13]. In this way, divided attention, memory recall, and motor training occur simultaneously and promote dual task practice. Studies support VR’s positive impact on gait, balance, and cognition [14], and its benefit on everyday life skills [10]. Despite these findings, systematic reviews on balance and gait performance outcomes are mixed [15, 16]. Past VR and treadmill (TM) walking studies found significant improvements in gait speed, walking performance, and obstacle negotiation [11, 17, 18], and a significant reduction in fall incidence [11] but these studies were conducted in largely neurologic populations (Parkinson’s disease (PD), multiple sclerosis (MS), stroke) and may not reflect community-dwelling Veteran outcomes. This pilot study investigated the feasibility and impact of non-immersive VR motor-cognitive treadmill training (VR-TM) on balance, walking, quality of life (QOL) and fall reduction in older Veterans with a history of falls or a documented fall risk. We hypothesized that VR-TM would be feasible and improve post-training balance, walking, QOL and fall incidence.

## Methods

This was a single-arm prospective pilot study. Due to its pilot nature, there was no randomization, and all participants received the same VR-TM training (GaitBetter, Inc., Rockville, Maryland) twice a week to complete 14 visits. Outcome measures were conducted pre- and post-training by an experienced assessor who was unblinded to the study training and was involved in the intervention. Fall incidence data was collected retrospectively prior to training, and prospectively during training sessions and over a six-month retention period. The study was approved by the University of Maryland Baltimore Institutional Review Board (IRB) and the Baltimore Veterans Affairs Research and Development Committee and study procedures were conducted in compliance with all ethical practices and guidelines. Because it was a nonrandomized, nonblinded pilot study available only to existing patients at a single medical center, it was not registered at ClinicalTrials.gov. Participants were enrolled if they met the inclusion and exclusion criteria (Table 1) and achieved a 10 out of 12 score on the evaluation to sign the informed consent form prior to study consent and enrollment.

**Table 1:** Study inclusion and exclusion criteria

Inclusion criteria	Exclusion criteria
Age 65 to 88 years old	Cognitive impairment such that the participant cannot understand the study requirements to answer the informed consent evaluation tool
Able to provide written consent	Acute or ongoing unstable medical conditions (e.g. angina, fracture or surgery)
Able to walk independently (may use assistive device) > 2 min	Pain (> 7 out of 10) in legs when walking
Stable medical condition and medication regime for 1 month prior to enrollment	Proliferative diabetic retinopathy or uncorrected vision resulting in inability to see simulated objects on virtual reality projection screen
History of falls, concern of falling or restricted activity due to fear of falling in the past year or diagnosed high fall risk conditions (e.g. diabetes, osteoarthritis)	

### Participants

**Fig. 1:**
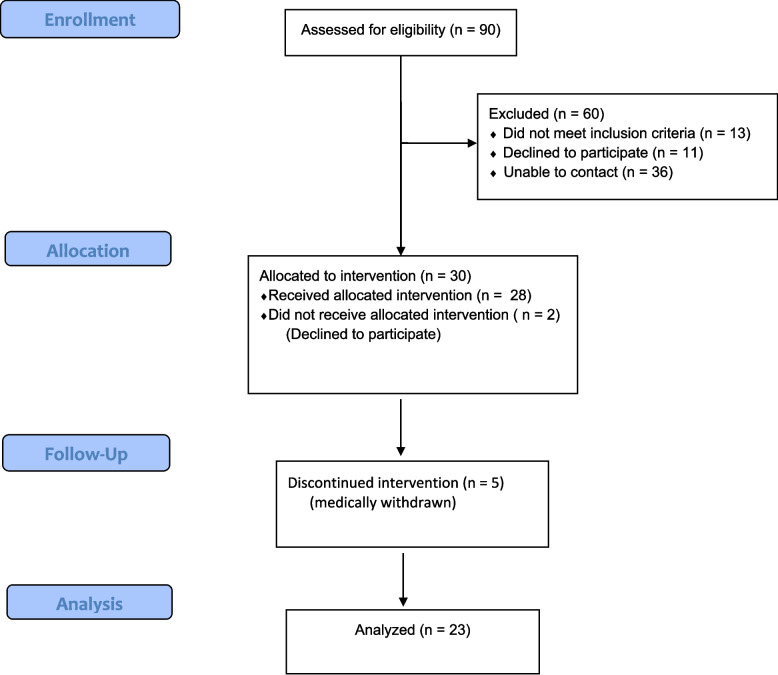
Study enrollment flow diagram

Ninety older adult Veterans from within the VA Maryland Health Care System (VAMHCS) were screened for eligibility and thirty met enrollment criteria (Fig. 1). Participant characteristics are detailed in Table 2.

**Table 2:** Demographic characteristics

Variable (unit)	All Participants *n* = 30	Final Analysis *n* = 23
Age (y)	74.3 (± 6.1)	75.3 (± 6.1)
Sex		
Male	26 (87%)	21 (91%)
Female	4	2
Ethnicity		
Black	18 (60%)	14 (61%)
White	8 (27%)	6 (27%)
Asian	1 (3%)	1 (4%)
Hispanic	1 (3%)	1 (4%)
Native American	2 (7%)	1 (4%)
Lives alone	15 (50%)	9 (39%)
Cognitive deficit (SLUMS score ≤ 26 points)	23 (77%)	19 (83%)
History of falls in past 6 mo	15 (50%)	12 (52%)
Self-report fear of falling	24 (80%)	20 (87%)
Falls Efficacy Scale (score > 70 indicates fall risk)	23.9 (± 16.1)	21.7 (± 13.1)
BMI (kg/m2)	29.5 (± 4.0)	29.0 (± 3.4)
Diagnosed comorbidities	16.8 (± 7.5)	15.4 (± 7.9)
Arthritis	24 (80%)	19 (83%)
HTN	22 (73%)	18 (78%)
Diabetes	17 (57%)	13 (57%)
Cardiac disease	12 (40%)	8 (35%)
CVA	7 (23%)	4 (17%)
Neuropathy	9 (30%)	8 (35%)
LE sensation impairment	21 (70%)	16 (70%)

Values are Mean (SD) or count (percentage)

*Abbreviations*: *SLUMS* St. Louis University Mental Status Exam, *BMI* Body Mass Index, *HTN* Hypertension, *CVA* Cerebral Vascular Accident, *LE* Lower Extremity sensation impairment reflects any deficit in L4-S1 dermatomes, toe proprioception or foot monofilament testing

### Initial assessment

Medical charts of potential participants were screened by study staff and reviewed by a geriatrician to determine eligibility. Following the initial eligibility screening and the informed consent process, participants completed a general physical examination, a cognitive screen using the St. Louis University Memory Scale (SLUMS) [19], lower extremity (LE) manual muscle strength tests and a sensation battery. The sensation battery included L4-S1 dermatome testing, toe proprioception and foot protective sensation via monofilament testing to account for pre-existing comorbidities impacting LE sensorimotor function (e.g., diabetes, peripheral neuropathy, lumbar spine arthritis). This first visit concluded with a five-minute TM walking test using a safety harness to confirm comfort and safety while walking at a self-selected speed. Baseline balance and walking performance outcomes, QOL and fall surveys were completed on a subsequent day (see Outcomes below).

### VR gait training system

**Fig. 2:**
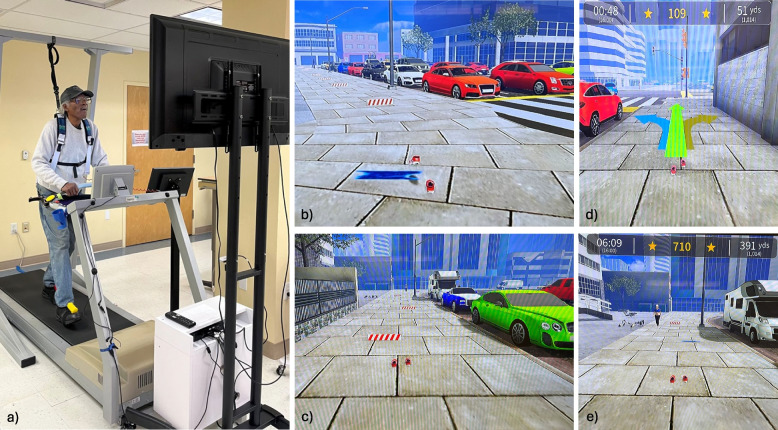
VR-TM motor-cognitive training Legend: The VR-TM system **a** consisted of a treadmill, removable foot markers, a motion camera system, a TV screen and safety harness that simulated gamified walking environments. Motor challenges included obstacle negotiation and in **b** the participant had to increase his step height to clear the hurdle and **c** increase his step length to clear the puddle. Cognitive challenges included **d** decision making to turn left, right or go straight toward a pre-set destination and **e** manage distractions such as people or traffic passing by

The VR-TM system used in this study was previously used in neurological conditions with positive outcomes [11]. Briefly, the system simulates walking environments using a two-dimension screen thereby preserving a general sense of presence in the real-world to minimize any cybersickness effects. Participants wore removable markers on their shoes while walking on the TM (Biodex Medical Systems Inc., Treadmill Model 950–420) and a camera-based motion capture system generated an avatar of their feet projected in real-time on a TV screen in the gamified VR environment (Fig. 2). Dual task walking, inclusive of obstacle negotiation, decision-making and memory tasks were safely provided in pre-defined VR walking scenarios (city street or park). Physical challenges included stepping over hurdles to promote step height and over puddles to promote step length. Cognitive challenges included memory recall, navigation, visual scanning, and audio and visual distractors (cars, pedestrians, fog). Immediate visual and auditory feedback enhanced knowledge of performance on step length, gait symmetry and obstacle clearance and knowledge of results were provided through cumulative scores at the end of each walking bout. The supervising trainer provided additional verbal cues on step timing and gait quality in weeks one and two that were tapered and removed in successive sessions. Participants completed all training sessions wearing a safety harness without body-weight support (Biodex Medical Systems Inc., Dynamic Unweighting System Model 945–470; or GaitBetter Inc., Suspension Bridge Safety harness).

The VR-TM training had 15 difficulty levels and advanced to higher levels based on participant performance. Participants started at level one and an adaptive algorithm adjusted the difficulty where > 80% success over the obstacles progressed the user to the next level and < 40% regressed them to the previous level. The pre-programmed VR parameters increased cognitive load through adjustments in walking duration, environmental settings, visual clarity, number of intersections, distractors, and obstacle type, size, and location (left or right). In this manner, participants had to be vigilant and adapt their walking pattern to clear the obstacles because sidestepping or walking around them was not an option on the TM. Participants walked at 80% of their self-selected TM walking speed in the first three visits to acclimate to the VR-TM training. Motor demand was advanced over the course of training by increasing TM speed at set intervals to achieve 40% above their self-selected walking speed with less hand support (double to single to none (if able)) by visit-14.

### Training

VR-TM sessions were provided in the study lab twice a week to complete the 14-visit protocol in seven weeks; allowances were made for missed visits and training did not exceed 11 weeks. Each session was supervised by an experienced research member, and participants were seen one-on-one in the first week and then concurrently with one other participant. Sessions lasted approximately 45-min and included two walking bouts with rest periods in-between. The initial two walking bouts were 10-min each and progressed to two 18-min intervals over the course of training. A safety harness ensured safety but did not provide body-weight support. Participants rated their training intensity using a 0–10 Borg Rating of Perceived Exertion (RPE) scale after each walking bout and rest breaks were approximately 5-min long.

### Outcomes

All outcome measures were completed by the same experienced assessor at the baseline pre-training visit and repeated upon completion of 14 training visits. This individual was involved in the training and unblinded to intervention data.

#### Balance

Balance assessments included the Mini Balance Evaluation Systems Test (Mini-BESTest), Four-Square Step Test (FSST), and the Standardized Walking Obstacle Course (SWOC). The Mini-BESTest is a clinical balance assessment used to identify balance performance in anticipatory, reactive, and dynamic gait control. It is a 14-item test that is reliable and valid in older adults and has a maximum score of 28 and a minimal clinically important difference (MCID) of four points [20]. A higher score is better, and scores < 23 indicate a fall risk in community dwelling older adults 70 to 79 years old [21]. The FSST is a measure of standing balance and ability to change directions. The FSST assesses the ability to step and clear obstacles while stepping forward, back and sideways as fast as possible over canes set on the floor designating four separate squares. The participant completed two timed trials and the fastest time was recorded. It is a reliable test in older adults and a FSST time < 15 s indicates a fall risk [22]. The SWOC is a reliable functional balance and mobility measure in older persons and stroke [23]. It consists of a 12.2-m long, 0.9-m wide curved pathway incorporating sit-to-stand transfers, obstacle avoidance, directional turns, walking on different surfaces and is completed under different environmental conditions including carrying a basket and low light. Participant completion time, step count, steps off the path, and number of stumbles were measured.

#### Walking

Walking assessments included the 2-Minute Walk Test (2MWT), Timed-Up and Go (TUG) and TUG Dual Task (TUG-DT). The 2MWT measures ambulatory function and endurance. Participants used an assistive device as needed and were asked to cover as much ground as possible in the two-minute timeframe [24]. The TUG is a timed performance measure of functional mobility and balance and is a valid and reliable measure of fall risk in older adults [25]. The test includes standing from a chair, walking 3-m, turning around and returning to a seated position in the chair. A faster time indicates better function and an older adult who takes > 13.5 s to complete the test is at risk for falls [25]. The TUG-DT is a valid and reliable modification that examines cognitive load. The test requires the participant to perform the TUG while counting backwards by three’s from 100. Dual task cost is the percent decline in performance under the dual task condition compared to the single task condition and is calculated as follows: ((TUG dual task -TUG)/TUG) * 100 [26].

#### Quality of life

The RAND 36-Item Short Form Health Survey (SF-36) is a reliable and valid standardized self-report measure of health-related QOL. It consists of 36 questions within eight domains of health that yields component physical and mental health summary scores. A higher score (0–100 max) reflects a greater ability to function and a more positive health perception [27, 28].

#### Falls

The Activities-specific Balance Confidence (ABC) Scale and the Falls Efficacy Scale (FES) measured confidence and concern about falling. The ABC Scale is a 16-item subjective measure of confidence performing household and community activities without a loss of balance or a sense of unsteadiness. It is a reliable measure of fear of falling in older adults where lower scores indicate greater fear, and higher scores indicate greater confidence (less fear) [29]. An ABC score ≤ 67% indicates a fall risk [30] and the MCID is 18.1% [31]. The FES is a 10-item self-report of confidence performing household activities. A lower score indicates greater confidence and a score > 70 indicates a fear of falling [32]. Fall incidence data was based on retrospective self-reported events six-months pre-enrollment, and prospectively during the intervention, and at monthly post-training phone calls over a six-month period. A fall was defined according to the World Health Organization as an event which results in a person coming to rest inadvertently on the ground or floor or other lower level [33].

#### Feasibility and satisfaction

Feasibility was evaluated based on retention, training adherence and satisfaction. Retention was determined by calculating the percentage of participants who completed the 14-visit protocol. Training adherence was evaluated based on the average weeks to complete the protocol and tolerance of the training bouts (number of rest breaks and adverse events (AE)). AEs were defined as any event that occurred during the course of the research study that either caused physical, social, economic, or psychological harm, or increased the risk of physical, social, economic, or psychological harm, or resulted in the loss of privacy and/or confidentiality to a research participant. At training sessions, participants were queried about any adverse events and specifically asked about any recent falls since the previous training session. These events were documented as appropriate. AEs and falls during the 6-month retention period were documented during monthly phone calls. Satisfaction was based on a satisfaction survey completed at the end of training using a 5-item Likert scale (1 = highly dissatisfied; 5 = highly satisfied) to score satisfaction with 1) having the VR-TM intervention as a part of their exercise program and 2) the challenge of the VR-TM training. Participants used a 10-item Likert scale (1 = not likely at all; 10 = extremely likely) to score their likelihood to recommend the training to another Veteran.

### Statistical analysis

In the absence of a similar study population to calculate sample size, we selected a sample size of thirty participants as a reasonable feasibility target. Data was collected from August 2022 to March 2025. Seven participants were excluded due to incomplete data resulting in twenty-three participants in the analysis. The data was examined for normality and descriptive statistics were extracted for all outcome measures. The change (post–pre) for each outcome was tested to see if the change was significantly different from zero. Pearson correlation coefficient assessed the relationship between change in ABC, Mini-BESTest, and number of falls. Prior to formal analysis, the data distribution was visually checked using histograms. Student’s t-test assumes the data are normally distributed, however in our small sample, some of the change scores appeared symmetric and several were decidedly non-symmetric. As a result, all the data could not be properly analyzed using a Student’s t-test, but all could be analyzed using a Wilcoxin test. A single analysis strategy using the Wilcoxon test was selected. A one-sample Wilcoxon test, as implemented in the R language (version 4.5) function exactRankTests (version 0.8.35) [34] was used. The exactRankTests function computes exact conditional *p*-values using the Shift-Algorithm of Streitberg and Röhmel [35] and the algorithm allows for ties in the changes.

## Results

Participant characteristics such as age, gender, ethnicity, home setting, fall history and comorbidities are reported in Table 2. Seven out of the 30 consented participants were withdrawn (attrition rate 23.3%). Two were withdrawn following consent and prior to interventional activities; one due to loss of interest and one due to inability to re-contact. Of the 28 participants who started the intervention, one withdrew due to personal issues, three due to medical issues unrelated to the study, and one due to increased knee pain with training. Twenty-three (76.6%) completed the study and were included in the study analysis. This cohort was primarily male (91%), non-white (74%), and 52% reported a fall six-months prior to study enrollment. Healthwise, they were overweight (BMI 29.0 kg/m^2^), had ten or more diagnosed comorbidities (65%), and 70% presented with lower extremity sensation deficits. All participants lived in the community; 39% lived alone, 52% used a walking device and 83% had cognitive deficits based on the SLUMS assessment. The baseline Mini-BESTest and ABC scores indicated a fall risk, and the FSST and TUG times approached fall risk thresholds of > 15 s and > 13.5 s, respectively. The 10-item FES [32] did not indicate a fall risk, however, 87% of participants reported a fear or concern of falling at baseline.

### Balance and walking

Table 3 depicts the outcome measure results. Post-training, Mini-BESTest scores increased from 20.91 points (SD = 4.20) to 23.13 (SD = 3.03) and showed significant improvement (*P* = .01). The largest gain occurred in the dynamic gait subcomponent with 16 participants (69.6%) crossing the 23-point fall risk threshold [21] after training compared to only nine (39%) pre-training. Six participants had a MCID post-training improvement of ≥ four points and four of these six had baseline LE sensation, proprioception and foot protective sensation deficits. There was a trend for improvement in the FSST (*p* = 0.06) and the average post-training time improved to 13.06 s (SD = 6.74) which is below the fall risk threshold of > 15 s [36]. A similar trend was observed in the TUG-DT (*p* = 0.08) with a decrease in performance time of −2.71 (8.20) seconds; however, the dual task cost was not significant (*p* = 0.16). The baseline 2MWT distance was well below established norms for men 75-to-79 years old (157.6 m) and post-training values improved but did not approach minimum detectable change [24].

**Table 3:** Outcomes and comparisons (*n* = 23)

Outcomes	Pre-training	Post-training	Change	*P*-value
Mini-BESTest (max score 28)	20.91 (4.20)	23.13 (3.03)	2.22 (2.70)	**0.01**
Four Square Step Test (s)	14.79 (12.51)	13.06 (6.74)	−1.73 (7.61)	0.06
Standard Walking Obstacle Course-Dimly lit room (s)	19.22 (14.24)	17.3 (0.49)	−1.91 (8.33)	0.41
2-Minute Walk Test (m)	127.33 (51.51	130.02 (46.33)	2.69 (24.73)	0.69
Timed Up and Go (s)	13.18 (5.60)	12.14 (4.02)	−1.05 (3.28)	0.10
Timed Up and Go Dual Task (s)	18.77 (10.17)	16.06 (6.72)	−2.71 (8.20)	0.08
Dual Task Cost (%)	41.08 (47.08)	31.44 (31.14)	−9.64 (37.56)	0.16
ABC (%)	65.18 (21.68)	73.04 (18.40)	7.86 (14.67)	**0.02**
FES	21.70 (13.08)	18.30 (11.03)	−3.39 (6.56)	0**.**35
SF36-physical	51.28 (19.46)	52.34 (19.61)	1.06 (15.05)	0.69
SF36-mental	53.79 (22.02)	55.05 (24.79)	1.26 (16.50)	0.87
SLUMS	22.48 (4.05)	21.96 (2.69)	−0.52 (3.68)	0.50

Values are means (standard deviation). *P*-value based on Wilcoxon signed rank test (modified to allow for exact matches). Bold values indicate statistically significant differences. *P*-value < 0.05

*Abbreviations*: *ABC* Activities Specific Balance Confidence Scale, *FES* Falls Efficacy Scale, *Mini-BESTest* Mini Balance Evaluation systems Test, *SF-36* Rand 36-item Short Form Health Survey, *SLUMS* St. Louis University Memory Scale

### Falls

Post training balance confidence (the ABC scale) significantly improved (*P* = .02). The mean ABC of 73% (≥ 67% threshold) placed participants above the fall risk threshold [30] and four participants had a > 18% gain reflective of a MCID [31]. There was no significant association between improved ABC and Mini-BESTest (*r* = 0.22); improved ABC and number of falls (*r* = −0.12); or improved Mini-BESTest and number of falls (*r* = 0.39).

The pre-intervention fall data was collected retrospectively, whereas the intervention and six-month retention fall data was collected prospectively which limited comparisons. Overall, ten participants self-reported at least one fall in the six months prior to study enrollment. Total reported falls during this time were 22 and five participants reported more than one fall. Eight participants fell during the 7-week intervention period and half were multiple fallers at enrollment. Of the eight falls, six experienced one fall and two with a history of multiple falls had multiple falls, which accounted for a total of 12 falls (10 at home; 2 in the community) (Table 4).

**Table 4:** Number of falls throughout study phases (*n* = 23)

Reason for Fall	**Study Phase**			
	**Pre-Intervention (6-months)**		**Intervention (7 weeks)**	**Retention (6-months)**
Trip/Slip				
Home		3		11
Community		1		1
Total		4		12
Obstacle Navigation				
Home		3		2
Community		1		4
Total		4		6
Reaching/Bending				
Home		2		2
Community		0		0
Total		2		2
Medical Reason				
Home		1		2
Community		0		1
Total		1		3
Alcohol Use				
Home		1		0
Community		0		0
Total		1		0
Cannot recall		0		2
Total	**22**	**12**		**25**
Total falls per month	**3.66**	**6.86**		**4.17**

Pre-intervention, intervention and retention phase fall data collection methodology differed and limit fall-related comparisons. Pre-intervention fall data is retrospective; intervention data is prospective and collected during the 7-week (1.75 month) training period and retention data is prospective and collected monthly during the 6-month post-training period

**Fig. 3:**
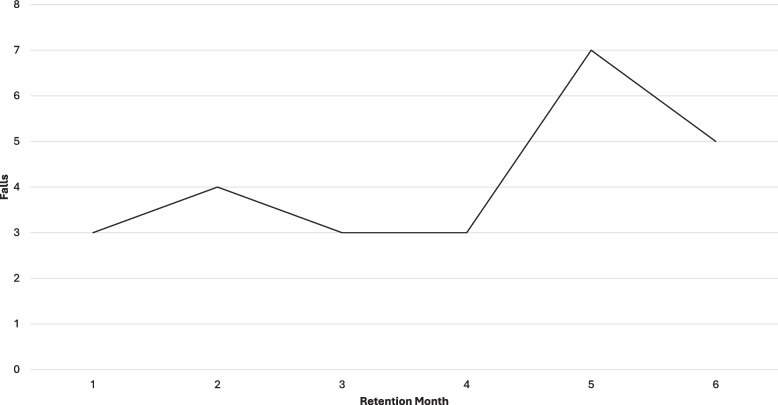
Number of falls during 6-month retention period Legend: The number of falls reported monthly during the 6-month retention period were generally stable in months one to three and increased after month four

Four falls occurred due to trips/slips, four during obstacle navigation (stairs), two from bending/reaching, one from dehydration, and one from alcohol consumption. None of these falls resulted in any missed training sessions, invasive treatment or major injury. During the 6-month retention period, 13 participants reported a fall, accounting for a total of 25 falls (17 at home; 6 in the community; 2 with unknown details). Eight participants experienced only one fall during this period, while five reported multiple falls. Four of these five were individuals who reported multiple falls six-months prior to study enrollment. Twelve falls occurred due to trips/slips, six during obstacle navigation (5 stairs/curb; 1 stepping over the dog), two while bending/reaching, three from medical issues (dehydration/leg pain) and two falls for which participants could not recall the details of their fall. None of these falls resulted in any invasive treatment or major injury. In totality, 78% of all falls (29 of 37) occurred at home and falls during the 6-month retention period increased over time (Fig. 3).

### Feasibility and satisfaction

Twenty-eight of the enrolled 30 participants progressed to training and twenty-three out of the twenty-eight (82.1%) completed the 14-visit protocol within 7.7-weeks and attended 99.1% of total sessions scheduled (13.9/14) (Table 5).

**Table 5:** Training adherence and outcomes

	Completers (*n* = 23)	Drop outs (*n* = 5)	Total (*n* = 28)
Ave Sessions Attended (out of 14)	13.9	5.2	12.32
Attendance Rate (%)	99.1	37.1	88
Progressed to no hand support on treadmill (n)	5	0	5
RPE (0–10)	3.76	4.64	3.86

Twenty-eight out of the 30 consented participants proceeded to the training phase

The average Borg perceived exertion score was 3.76 out of 10 (moderate-to-sort of hard) and five participants required additional one to two minutes breaks during their training: two in the first few weeks; two in the last few weeks; and one throughout the training period. Overall, participants rated their training satisfaction as a 4.7 out of 5 and 94% would recommend the VR-TM training to a friend rating it 9.8 out of 10. Training adverse events were minor and included: 1) a bruised knee when attempting to clear a virtual obstacle (knee lifted too high and hit the treadmill handrail and 2) exacerbation of chronic knee osteoarthritis resulting in withdrawal after two-weeks of training.

## Discussion

This pilot study examined the acceptance and effect of non-immersive VR treadmill training on fall risk and fall incidence among community-dwelling older Veterans with a fall history or a documented fall risk. The training was feasible based on adherence, acceptance and high satisfaction rates and was without serious adverse events. Participants had improved Mini-BESTest scores post-testing; particularly on the dynamic gait stability component but no other evident improvements were identified and the number of falls six-month post-training did not show a reduction. Balance confidence on the ABC scale increased but no evident improvements were identified in outcomes for walking performance, walking endurance, or QOL measures post-training. This 14-visit 7-week VR- TM program saw no significant change in fall incidence six-months after completion, though this would not be expected in such a small heterogeneous cohort.

### Balance and fall risk reduction

Significant improvement on the Mini-BESTest reflects improved balance and a reduction in fall-risk factors. The Mini-BESTest assessed six distinct balance components and included anticipatory postural control, reactive responses, sensory orientation, and dynamic gait stability [21]. On average, participants had the largest gain in the dynamic gait stability component. This positive finding is consistent with our gait focused VR-TM training; however, no significant TUG and TUG-DT performance change was detected despite improvements in the correct direction. This is inconsistent with previous VR-TM studies where significant TUG time improvement occurred after a 15-visit 5-week clinical program [18], and after an 18-visit 6-week study [17] in neurologically heterogenous patients and individuals with MS, respectively. Our contrary results may be due to differences in the populations studied and our unexpected high enrollment of participants with pre-existing sensorimotor, arthritic and cognitive deficits. Unlike previous studies, our community-dwelling Veterans had sensation and musculoskeletal deficits inclusive of peripheral neuropathy (35%), arthritis (83%) and a high percentage of MCI (83%). The TUG and the TUG-DT test mobility during a multi-step functional task wherein the TUG is the task itself, and the TUG-DT has an added cognitive load of serial subtraction. As such, each assesses physical and cognitive ability within the context of a daily activity that incorporates sit-to-stand, walking, and turning. Studies examining sit-to-stand performance alone found lower limb proprioception, tactile sensitivity, balance, LE strength and psychological factors to be significant and independent indicators of performance time [37]. The VR-TM training offered sensorimotor, balance and general strengthening during repetitive stepping but was without specific lower extremity strength training. The combined physical and cognitive deficits identified in our Veteran cohort may have limited available resources to improve TUG and TUG-DT time and further reduce that aspect of fall risk in the context of life activities. Future studies may evaluate whether different measures of dual task performance are better suited to assess change for this population.

### Walking and obstacle negotiation

The VR-TM system’s unique gaming environment targets motor-cognitive training through divided attention, motor planning and reaction time while negotiating a virtual obstacle course. As a result, past studies applying this system [17, 18, 38] found improvement in obstacle negotiation while walking. Contrary to these findings, our participants had modest FFST and SWOC gains without significant post-training improvement. In aging, evidence supports combined motor and cognitive training over long periods of time to improve cognitive function [39] and similarly; at least 52 h of exercise is needed for associated cognitive improvement in older adults with and without cognitive impairment [40]. An examination of VR-TM study duration in community-living older adults with PD and without MCI found cognitive improvements after 12-weeks of training not seen in the 6-week training group [41]. This same study found cognitive improvements were retained up to 1-month post-intervention, but not by the end of the six-month retention period. Interestingly, the multi-site parent study conducted by Mirelman et al. 2016 [11] found less consistent post-training obstacle clearance, mobility, endurance and QOL effects in a subgroup (*n* = 43) of participants with MCI after 6-weeks of VR-TM training [11]. A training period longer than 14-visits may be required to improve cognitive processes for obstacle negotiation; however, modest improvements are difficult to demonstrate in our heterogenous population without a larger sample size.

### Fall incidence

Self-perceived balance confidence improved post-training; however, compared to fall recall data at baseline, the incidence of reported falls after training did not decrease and was higher six-month post-training than pre-training. Though a statistically significant improvement in fall rate was not expected in this small cohort, an apparent increase was also unexpected and contrary to previous studies where fall incidence and fall rate decreased [11, 42]. The discrepancy may be related to our enrollment criteria, duration of training and limitations in historical self-report. Our enrollment criteria included those with a fear of falling (FoF) that may not have had a fall in the previous six months whereas previous VR-TM studies only included those with multiple falls [41]. FOF and the number of previous falls are predictors of falls in older, community-dwelling adults [43]. However, the relationship between FoF and fall occurrence varies based on fall history [43]. Those with multiple previous falls have lower association of FoF with fall rate when compared to single fallers or non-fallers. Thus, previous studies may have found significant reductions in fall incidence due to participants having a higher fall rate pre-intervention than our population. Additionally, previous research found a greater reduction in falls with 12-weeks of VR-TM training when compared to 6-weeks [41]. Improving or verifying fall recall at baseline would be difficult, but perhaps a longer intervention in a larger cohort would better assess whether this intervention can reduce subsequent fall rate in this study population.

The incidence of falls reported varies based on retrospective or prospective data collection, with prospective reporting (i.e. fall calendars) being the gold standard [44]. Freiberger and colleagues [44] showed the number of falls reported retrospectively during the previous 12-months were less than prospective reporting during that same timeframe (45.9% vs 56.9%, respectively). This difference was more pronounced when reporting two or more falls (37.8% vs 17.2%). We do not have specific data to answer this question, but it is possible that this predominantly male cohort of Veterans with a high prevalence of MCI underreported “how many falls in the past six months” at enrollment but were less prone to underreport on subsequent monthly telephone assessments. In our study, 13 participants denied a fall six-months prior to enrollment but 31% of these non-fallers sustained a fall while enrolled. Our pre-intervention fall data may have underestimated the number of falls (collected retrospectively via historical self-report), whereas our retention data was more accurate due to utilizing the gold standard reporting with monthly fall calendars and phone calls. The potential underreporting of falls pre-intervention may have impacted the lack of a significant reduction in falls seen.

Our study design cannot discern between these factors but provides important information on the most common fall location and type. Most falls occurred in the home setting and were associated with trips/slips or obstacle negotiation errors; thereby supporting the importance of step clearance and obstacle negotiation as a fall prevention target. Whereas VR-TM training was successful in reducing an aspect of fall risk on the Mini-BESTest it did not reduce the number of falls over a six-month retention and many falls were obstacle related. Interestingly, fall rates remained steady in the first four months post intervention suggesting a potential protective effect in these early months.

### Feasibility

While not specifically assessed, our cohort had a high disease burden and their baseline BMI and 2MWT indicated a lack of regular exercise. Engaging in greater amounts of physical activity and exercise can reduce cognitive decline, [45] improve balance and decrease fall risk [46]. Effectiveness is reliant on adherence [46] and unfortunately, only 13.9% of community-dwelling older adults meet the Center for Disease Control and Prevention (CDC) aerobic activity and muscle strengthening guidelines and this percentage drops to 7.8% among individuals with four or more chronic conditions [46]. One advantage of VR is its ability to increase exercise motivation and promote higher exercise duration [47] and intensity [48]. Consistent with these findings, over 76% (23/30) of our participants completed the training at intensity levels at or above a moderate BORG rating without serious event or injury. This training retention is comparable and slightly higher than other VR FPEP [49] and traditional community-based FPEP [50]. Evidence supports non-immersive VR’s low rate of cybersickness, motivational gaming features, and compliance as a viable rehabilitation method in older adults [51]. Overall, our findings concur, and non-immersive VR-TM training was well tolerated at a moderate intensity without serious adverse events supporting safety, feasibility and potential for a higher intensity or longer duration protocol. To date, VR studies targeting aerobic or cardiovascular changes in older adults are small and varied [52, 53] and the requisite dose is unknown.

In conclusion, VR-TM was feasible among a heterogenous group of community-living Veterans at risk for falls, with no unexpected or serious adverse events attributable to the intervention. For most participants, it was well tolerated at a moderate intensity and enjoyable enough that they would recommend it to others. Our results expand the application of VR-TM training to a medically complex older adult population. These positive findings support the potential of a higher intensity or longer duration training to address risk and fall prevention in this group.

### Limitations

Study limitations include the small sample size based on a feasibility target versus a calculated sample sized, our single-arm intervention design that lacked a control group, varied fall data collection (retrospective at enrollment and prospective throughout study) and a potential outcome bias due to assessments by an unblinded assessor involved in study training.

## Data Availability

The datasets supporting the findings of this study are available on request from the corresponding author [SC]. The data are not publicly available due to containing information that could compromise research participant privacy/consent rights.

## Abbreviations

FPEP: Fall prevention exercise program
VR: Virtual reality
TM: Treadmill
PD: Parkinson’s disease
MS: Multiple sclerosis
VR-TM: Non-immersive virtual reality motor-cognitive treadmill training
QOL: Quality of life
VAMHCS: VA Maryland Health Care System
SLUMS: St. Louis University Memory Scale
LE: Lower extremity
Mini-BESTest: Mini balance evaluation systems test
FSST: Four square step test
SWOC: Standardized walking obstacle course
MCID: Minimal clinically important difference
2MWT: Two-minute walk test
TUG: Timed up and go test
TUG-DT: Timed up and go dual task test
SF-36: Rand 36-item short form health survey
ABC: Activities-specific balance confidence scale
FES: Falls efficacy scale
AE: Adverse events
MCI: Mild cognitive impairment
FOF: Fear of falling
BMI: Body mass index
PA: Physical activity
CDC: Center for Disease Control and Prevention
BORG: Borg rating of perceived exertion scale
